# 

**Published:** 1860-03

**Authors:** 


### Reviews and Bibliography.

Several notices prepared for this section have been unavoidably deferred.

